# Distinct Insular Functional Connectivity Changes Related to Mood and Fatigue Improvements in Major Depressive Disorder Following Tai Chi Training: A Pilot Study

**DOI:** 10.3389/fnint.2020.00025

**Published:** 2020-05-28

**Authors:** Anna Xu, Chloe S. Zimmerman, Sara W. Lazar, Yan Ma, Catherine E. Kerr, Albert Yeung

**Affiliations:** ^1^Department of Cognitive, Linguistic, and Psychological Sciences, Brown University, Providence, RI, United States; ^2^Department of Neuroscience, Brown University, Providence, RI, United States; ^3^Alpert Medical School, Brown University, Providence, RI, United States; ^4^Department of Psychiatry, Massachusetts General Hospital, Boston, MA, United States; ^5^Harvard Medical School, Boston, MA, United States; ^6^Center for Dynamical Biomarkers, Division of Interdisciplinary Medicine and Biotechnology, Beth Israel Deaconess Medical Center, Harvard Medical School, Boston, MA, United States; ^7^Depression Clinical and Research Program, Department of Psychiatry, Massachusetts General Hospital, Harvard Medical School, Boston, MA, United States; ^8^Benson Henry Institute for Mind Body Medicine, Massachusetts General Hospital, Harvard Medical School, Boston, MA, United States

**Keywords:** major depressive disorder, Tai Chi, fatigue, vitality, insula, resting-state functional connectivity, interoception, mood

## Abstract

**Objective**: Tai chi (TC), a contemplative practice combining slow movements and deep breathing, has been shown to be clinically effective in alleviating depressive symptoms. Feelings of fatigue or low vitality often accompany major depressive disorder (MDD) though they are commonly overlooked and not well understood neurologically. By using resting state functional connectivity (rs-FC) using the insula as the seed, this study examines the relationship between mood and vitality symptoms in MDD and how they are impacted by TC training.

**Methods**: Patients (*N* = 16) with MDD participated in a 10-week TC intervention. Self-report scores of vitality (using the SF-36 scale) and depressed mood (using the Beck Depression Inventory) as well as rs-fMRI were collected pre- and post-intervention. A seed-to-voxel approach was used to test whether changes in insular rs-FC were related to therapeutic improvement in MDD-related symptoms resulting from TC practice.

**Results**: We found decreased self-reported depressed mood and increased vitality following the TC intervention. Furthermore, decreases in depressed mood were associated with increased rs-FC between the right anterior insula (AIC) and superior temporal gyrus and caudate (cluster-corrected *p* < 0.05). Increased vitality was associated with increased rs-FC between the right posterior insula (PIC) and regions associated with sensorimotor processes (cluster-corrected *p* < 0.05).

**Conclusion**: These results provide support for differential changes in insula connectivity as neural correlates of symptom improvement in MDD.

## Introduction

Major depressive disorder (MDD) is a pervasive and debilitating clinical disorder associated with mood-related symptoms, such as melancholy or anhedonia (Demyttenaere et al., 2005), and persistent attentional biases toward self-related information (i.e., rumination; Kaiser et al., 2015). Although the mood-related symptoms of MDD have been well studied, the prevalence of fatigue and other bodily symptoms in MDD are often overlooked (Demyttenaere et al., 2005). Fatigue, defined as a *decrease in the subjective sense of vitality* (Bjorner et al., 2007; Brown et al., 2011), imposes serious limitations on daily functioning, significantly impairing interpersonal and social relationships (Demyttenaere et al., 2005). Furthermore, fatigue is not readily alleviated by antidepressants (Belmaker and Agam, 2008). Although fatigue is one of the most commonly reported prodromal somatic symptoms in patients with their first major depressive episode (Demyttenaere et al., 2005), it is rarely targeted specifically in behavioral interventions for MDD. Because an enhanced sense of vitality is considered an indicator of improved fatigue (McHorney et al., 1994), the cultivation of vitality through behavioral interventions becomes directly relevant as a therapeutic target for MDD. A better mechanistic understanding of interventions that can enhance the sense of vitality in addition to typical mood-related symptoms is, therefore, necessary to elucidate the underlying neurophysiological alterations important in alleviation of MDD symptomology.

Tai chi (TC) may be one such clinical intervention that directly addresses both vitality and mood symptoms in MDD. Originating from China, TC is a mind–body contemplative exercise that facilitates the sense of vitality (i.e., *qi* or vital energy flow in the body) by incorporating awareness of posture; slow, focused, and low-impact movements; and deep breathing to improve underlying physiological and psychological imbalances (Wolf et al., 1997; Sandlund, 2000; Xiang et al., 2017). Previous studies have documented TC’s clinical efficacy in improving both the subjective sense of vitality as well as mood in a wide variety of clinical populations, including rheumatism (Uhlig et al., 2010), heart failure (Yeh et al., 2016), multiple sclerosis (Burschka et al., 2014), and cancer (Zeng et al., 2014) as well as in risk of falling (Del-Pino-Casado et al., 2016), Parkinson’s disease, cognitive impairment and dementia, stroke, and chronic obstructive pulmonary disease (Huston and McFarlane, 2016). Moreover, TC has increasingly been studied for its efficacy in treating MDD (Chou et al., 2004; Lavretsky et al., 2011), especially in regards to improving mood symptoms and sleep quality (Ma et al., 2018).

In recent years, TC has been considered a part of a larger class of meditative movement exercises, which include but are not limited to yoga, qigong, and other forms of movement from Western somatic practices (Forge, 2005; Tsang et al., 2008; Larkey et al., 2009). Meditative movement exercises are a category of exercise defined by: (a) some form of movement or body positioning; (b) a focus on breathing; (c) a cleared or calm state of mind with a goal of; (d) deep states of relaxation (Larkey et al., 2009). A recent systematic review of meditative movement practices found significant impact on depression severity (Zou et al., 2018). The therapeutic effects of meditative movement on affective disorders, such as depression and anxiety, seem to be related to the cultivation of awareness of internal bodily sensations (i.e., body awareness; Payne and Crane-Godreau, 2013), similar to findings with standard seated meditation studies (Segal et al., 2010; Segal and Walsh, 2016). Recent studies have begun to identify important subjective and physiological aspects of this body-awareness mechanism that are particularly relevant to an improved sense of vitality in MDD (Payne and Crane-Godreau, 2013).

Experientially, mood and vitality may be related to an individual’s ability to consciously sense the state of the body’s internal condition. This sensing capacity is often referred to as interoception (Mehling et al., 2011). Improvements in interoceptive awareness following meditative movement training is considered one mechanism by which these practices are able to facilitate therapeutic changes in a variety of clinical disorders (Payne and Crane-Godreau, 2013). This is relevant to MDD given that patients often exhibit impairments in accurately detecting interoceptive signals (Wiebking et al., 2010; Furman et al., 2013; Farb et al., 2015). Deficits in interoceptive processing may be related to the somatic symptoms of low vitality as well as to mood-related symptoms in MDD (Zou et al., 2018). Core aspects of TC practice certainly involve the training of interoceptive capacities through cultivating attention to the internal state of the body (Forge, 2005; Tsang et al., 2008; Burschka et al., 2014). A growing literature on meditative movement and meditation studies increasingly links enhanced interoceptive abilities with positive benefits for depressed populations (Farb et al., 2010, 2015) as well as reduction in depressive symptoms in other illnesses (Lilja et al., 2016; de Jong et al., 2018).

Neuroimaging studies related to interoception most frequently identify the insula as a primary neural correlate of interoceptive awareness (Critchley et al., 2004; Seth et al., 2011; Simmons et al., 2013). The insular cortex can be differentiated into multiple anatomical and functional regions associated with complex, multidimensional aspects of both MDD experience (Wiebking et al., 2010; Sliz and Hayley, 2012; Avery et al., 2014; Dutta et al., 2014) and interoceptive functioning (Craig, 2013; Strigo and Craig, 2016). It has been suggested that the anterior insula (AIC) is involved in emotional/salience appraisal of interoceptive stimuli and is a major node of the salience network (Cauda et al., 2011). The AIC is further involved in integrating representations of the outside world with the body’s internal state (e.g., in emotional experiences and self-awareness; Craig, 2009; Farb et al., 2013). The PIC, with its projections to primary sensory and motor cortices, has been implicated in sensorimotor integration and is considered by some to be a putative primary interoceptive cortex (Critchley et al., 2004; Cauda et al., 2011; Deen et al., 2011). Importantly, the insula propagates sensory signals about the state of the body and its integration with the external environment to higher-order prefrontal cortex (PFC; Critchley et al., 2004; Critchley, 2005; Mutschler et al., 2009; Seth et al., 2011), indicating a prefrontal involvement in interoceptive functioning necessary for understanding the neural network connections involving the insula. MDD pathology has been associated with alterations in insular activity with dorsal mid-insula and left AIC both showing diminished activity in the fMRI at rest and during interoceptive tasks and correlated with both depression severity and altered bodily perception (Wiebking et al., 2010, 2015; Wiebking and Northoff, 2015; Avery et al., 2014).

Although such studies reveal an involvement of insular activity and connectivity in MDD-related psychopathology, neuroimaging studies of mindfulness meditation training demonstrate that the AIC is also responsive to meditation training (Farb et al., 2013). Moreover, mindfulness training seems to be linked to greater negative connectivity between key nodes in the PFC related to rumination (e.g., medial PFC) and the PIC during interoceptive attention tasks (Farb et al., 2013, 2015). Importantly, such mindfulness studies indicate that the anterior and PIC both demonstrate plasticity following body-awareness practices (Farb et al., 2013). This meditation training–related plasticity and engagement of the insula may extend to meditative movement practices (Payne and Crane-Godreau, 2013), making it directly relevant to explore in the context of TC as a treatment for MDD. Few studies, however, have specifically examined how insular connectivity is associated with meditative movement–related improvements in MDD symptoms. Given that TC in particular involves the active movement of the body to circulate vital energy and changes in the internal state of the body, the insula may be an important neural hub associated with changes in overall mood as well as the bodily sense of vitality.

In recent years, a few neuroimaging studies have begun to examine TC-specific effects on the brain. fMRI studies have indicated that TC can have important impact on frontal cognitive control areas (Tao et al., 2016, 2017a,b). Moreover, resting-state functional dynamics of the brain appear to become more integrated in TC practitioners compared to non-practitioners (Wei et al., 2013, 2014, 2017). In the present study, given that TC is a form of meditative movement, our goal was to test whether findings regarding the impact of meditation-based interoceptive training on insular processing extended to TC and whether insular connectivity changes were related to improvements in vitality as a functionally relevant outcome in MDD symptom alleviation. No prior studies have directly examined the impact of TC training on the functional connectivity of the insula in MDD nor examined the relationship between the insula and the felt sense of vitality.

Using seed-to-voxel resting-state functional connectivity (rs-fc), we hypothesized that the connectivity of the insular cortex would be a key neural correlate of both subjectively ranked mood and vitality improvement following TC training and that these improvements would be anatomically differentiated within the insula. Specifically, we hypothesized the following:

(1) MDD patients would exhibit decreased connectivity between AIC and default mode network regions (e.g., medial PFC) post TC intervention related to improvement in mood. This would be consistent with findings related to meditation-related neural changes.
(2) Participants would exhibit enhanced rs-FC between PIC and AIC related to symptom improvements post intervention, reflecting increased flow of information from posterior to anterior insula and enhanced integration of physical sensations after TC.
(3) Increased vitality post intervention would be related to the connectivity of the PIC, reflecting increased awareness of sensory information and the flow of this information into the rest of the brain.

## Materials and Methods

### Participants

A total of 16 individuals born in China but currently living in the greater Boston area were recruited (10 females; age range of whole group: 46.5 ± 18.5, mean age = 54.5, std dev = 11.64). Most were referred to the study by South Cove Community Health Center, which serves Asian Americans in Boston, MA, USA. Subjects were recruited with advertisements and by referrals from South Cove’s primary care and mental health clinicians as well as routine depression screening at South Cove’s primary care clinics. Because TC was taught in Chinese languages in this study, only participants who spoke fluent Mandarin or Cantonese were enrolled to ensure understanding and to encourage social interaction and mutual support in class. The participants in this study are part of a larger pre–post assessment study of the effects of a 10-week TC intervention on sleep quality among patients with depression reported elsewhere (Ma et al., 2018).

All screening and testing materials were translated into both Mandarin and Cantonese, and all participant interaction was conducted in those languages. Potential subjects were phone screened by our bilingual research staff using an IRB-approved protocol that includes both a study-specific phone-screen questionnaire and the Patient Health Questionnaire-9 (PHQ-9). If patients met initial screening criteria, they were scheduled for an in-person screening visit to check if a patient met the inclusion criteria. The inclusion criteria included: (1) self-identify as being of Chinese ethnicity and fluent in Mandarin and/or Cantonese; (2) be 18–65 years of age; (3) satisfy DSM-IV-R criteria for MDD assessed by a psychiatrist using instruments including the Chinese bilingual version of the semi-structured psychiatric interview (SCID; CB-SCID-I/P); (4) have a baseline score of 14–24 on the Hamilton Depression Rating Scale (HAM-D-17); and (5) have had no regular (defined as ≥ 3 times/week for ≥ 2 months) TC training/practice or other forms of mind–body intervention in the past 6 months. Exclusion criteria included: (1) primary psychiatric diagnosis other than MDD; (2) history of psychosis, mania, severe cluster B personality disorders, or active alcohol or substance abuse/dependency disorders in the past 6 months; (3) unstable medical conditions as judged by investigators; (4) usage of or plans to use confounding treatments, including antidepressants and CAM treatments thought to have beneficial effects on mood, such as St. John’s wort, S-Adenosyl methionine (SAMe), omega-3 fatty acids, light therapy, conventional psychotherapy, mind–body interventions (e.g., yoga, mindfulness training, muscle relaxation training, etc.); (5) current active suicidal or self-injurious potential necessitating immediate treatment; (6) current pregnancy; (7) metallic implants; (8) claustrophobia; and (9) patients who have atrial fibrillation or an implanted pacemaker.

The study was approved by the institutional review board (IRB) of the Massachusetts General Hospital. Patients were scanned within 2 weeks before the TC intervention and within 2 weeks after the intervention. Batteries of behavioral questionnaires were completed by the participants at baseline, week 5, and week 10 of the TC intervention.

### Intervention

Classes were conducted by a TC master who had more than 25 years of training experience. TC participants received 1-h TC training sessions two times per week for 10 weeks. The instructor followed a standardized protocol, which included the first 24 of the traditional 108 movements of Yang-style TC. Participants were asked to practice TC at home with an instructional DVD at least three times per week and 30 min each time. At the end of 10 weeks, the participants were expected to be able to practice the 24 basic movements on their own. TC exercise logs were given to participants every week to record practice compliance and adverse events. Throughout the program, we tracked patient attendance at classes to ensure they were complying with the class and used practice logs to monitor their compliance with home practice. Research assistants contacted participants when they missed a class to assess barriers to participation and worked with the participant to overcome these barriers. Participants were interviewed using the HAM-D before and after intervention to assess changes in their levels of depression.

### fMRI Image Acquisition

All subjects were instructed to stay still throughout the scanning with eyes open and to blink naturally, stay awake, and not to think about anything in particular. Anatomical MP-RAGE scans were 6 min in duration while resting-state MRI scans were 6.7 min in duration.

All scans were conducted at the Massachusetts General Hospital Siemens TIM Trio 3T MRI scanner. For the resting-state scan, 200 T2*-weighted gradient echo echo-planar (EPI) images were collected using a BOLD technique (TR = 2,000 ms, TE = 30 ms, flip angle = 90°, scan duration = 6.7 min) for each participant. Thirty-seven interleaved whole-brain slices were acquired per image (slice thickness = 3 mm, FOV = 192 mm, matrix = 64 × 64, in-plane resolution = 3 × 3 mm). Images were collected along the transverse plane, parallel to the anterior–posterior commissure line.

Prior to functional image collection, high-resolution T1-weighted structural images were obtained from each participant using the magnetization-prepared rapid acquisition gradient echo (MP-RAGE) anatomical set (full brain = 176 sagittal slices, TR = 1900 ms, TE = 2.98 ms, flip angle = 9°, slice thickness = 1.0, FOV = 256 mm, matrix = 256 × 256, voxel size = 1 × 1 × 1 mm).

### Resting-State fMRI Preprocessing

Data analysis and image preprocessing were performed using the software Statistical Parametric Mapping (SPM12; Wellcome Trust Centre for Neuroimaging, University College London) *via* the CONN toolbox (Version 16.b) *default MNI* pipeline (Whitfield-Gabrieli and Nieto-Castanon, 2012). This pipeline consisted of: (1) functional realignment and unwarping, (2) slice-timing correction; (3) structural segmentation and normalization; (4) functional normalization to MNI; (5) outlier detection; and (6) smoothing. Slice timing correction followed as did spatial realignment and motion correction. Preprocessing included co-registration of functional scans with MP-RAGE structural scans, segmentation, realignment, normalization to the MNI atlas, and smoothing with an 8-mm kernel. A temporal band-pass filter of 0.01–0.1 Hz was applied, and residual head motion parameters were regressed out.

Motion correction was performed using the default configuration of the CONN toolbox. In particular, anatomical CompCor (Behzadi et al., 2007; Muschelli et al., 2014) extracted the first five principal components from white matter and the first five principal components from cerebrospinal fluid using a singular value decomposition. The CompCor model was augmented with spike regressors that flagged for censoring any volumes with translational motion in excess of 0.5 mm or rotational motion in excess of 0.05 radians (Power et al., 2012; Satterthwaite et al., 2013). Finally, six head motion estimates (three translational, three rotational) and their first temporal derivatives (computed as backward differences) were added to the model. The BOLD data were projected onto the column space of the model matrix and the residuals of the least-squares fit were retained for further analysis.

### Seed-Selection

Regions of interest (ROIs) were created based on previous studies that showed these regions to have domain-specific relevance to our hypotheses. For this study, we chose the AIC and PIC as ROIs due to their common role in interoceptive processing and their involvement in both depression studies as well as neuroimaging findings from other contemplative practices (e.g., meditation; Farb et al., 2013). We created all ROIs as 6-mm spherical seeds centered on the MNI coordinate of peak intensity. Our ROIs were defined anatomically using the online database Neurosynth^1^ to localize regions of interest. AIC was centered at MNI: 38, 20, −2, and the PIC was centered at MNI: 38, −12, 4. The negative of the *X*-coordinate was used as comparable coordinates on the opposite side of the brain (i.e., the left side)—coordinates for the left AIC were centered at MNI: −38, 20, −2, and coordinates for the left PIC were centered at: −38, −12, 4. All ROI masks were generated using WFU PickAtlas and run through the Statistical Parametric Mapping 8 (SPM 12) package.

### Statistical Analysis of Functional Connectivity

We utilized CONN toolbox (Version 16.b) 38 to calculate the significance and strength of Pearson correlations between the BOLD time course of our seed regions of interest and other voxels of the whole brain. These correlation coefficients were then converted to normally distributed scores using Fisher’s transform and used in second-level group analyses. In second-level group analyses, we first performed multiple general linear models to assess insula-related FC changes following TC. We next examined the association between differences in resting-state functional connectivity pre- and post-TC intervention and changes in symptom scores. In our first model, we entered changes in BDI scores as our between-subjects effect of interest, pre- and post-TC intervention as our between-conditions effect of interest, and an intercept term. Changes in BDI scores were calculated by subtracting post-intervention BDI scores and pre-intervention BDI scores. We further examined the relationship between BDI scores and insula-related FC pre-intervention and post-intervention to better unpack results related to changes in BDI scores.

In our next analyses, we entered changes in VT scores as our between-subjects effect of interest, pre- and post-TC intervention as our between-conditions effect of interest, and an intercept term. Changes in VT scores were similarly calculated by subtracting post-intervention score from pre-intervention score. Maps were then thresholded at a cluster-size FDR-corrected threshold of *p* < 0.05, voxelwise uncorrected height threshold of *p* < 0.001.

### Behavioral Measures

Beck Depression Inventory (BDI):

The BDI is a widely used four-point Likert scale, psychometric test for measuring depression severity. Each response contains a value of zero to three. Higher total scores indicate more severe depressive symptoms. The inventory contains a total of 21 questions inquiring about subjects’ feelings in the previous week (Beck et al., 1961).

Short Form Survey-36 Vitality Subscale (SF-36 VT):

The SF-36 is a widely used instrument to health-related quality of life. It consists of 36 items in eight scales: physical functioning (10 items), role limitations caused by physical health problems (four items), role limitations caused by emotional problems (three items), social functioning (two items), emotional well-being (five items), vitality (four items), pain (two items), and general health perceptions (five items). These eight scales can be aggregated into two summary measures: the physical component score (PCS) and mental component score (MCS). An additional single item assesses change in perceived health. Each scale is directly transformed into a 0–100 scale on the assumption that each question carries equal weight. Lower scores indicate more disability. For this study, we were specifically interested in the vitality subscale (McHorney et al., 1994).

### Behavioral Data Statistical Analysis

The behavioral data were analyzed using the statistics software SPSS 22. Results with *p* < 0.05 were considered statistically significant in our analyses.

To test differences between pre- and post-TC intervention within subjects, we conducted a paired-samples *t*-test for BDI scores and another for SF-36 VT scores. We also ran a Pearson’s correlation to examine the correlation between BDI score differences pre- and post-TC intervention with SF-36 VT score differences. To analyze whether VT scores predicted BDI scores, we ran a simple linear regression on measures that were significantly correlated in our Pearson’s correlation tests.

## Results

### Changes in Symptom Measures

Table 1 shows the descriptive statistics for the BDI and SF-36 VT scales. A paired-samples *t*-test revealed a significant decrease in BDI scores pre- and post-intervention (mean dif = −11.19; *t*_(15)_ = −3.52, sig. (two-tailed) *p* = 0.003, *d* = −1.6) as well as a significant increase in VT scores pre- and post-intervention (mean dif = 8.31; *t*_(15)_ = 2.707, sig. (two-tailed) *p* = 0.016, *d* = 1.41).

**Table 1:** Descriptive statistics of Beck Depression Inventory (BDI) and SF36-Vitality (VT) subscale scores pre- and post-Tai Chi intervention.

Week	Measure	Mean	SD	Range	*p*-value
0	BDI	22.31	9.1	5–37	0.003
10	BDI	11.13	10.58	0–28	
0	SF36-VT	42.71	7.64	30.1–56.2	0.016
10	SF36-VT	50.84	8.66	34.9–60.9	

*P-value indicates significance of differences between scores from Week 10 and Week 0 from paired-sample t-tests*.

To further analyze whether VT differences predicted BDI differences, a linear regression analysis indicated that SF-36 VT increases significantly predicted BDI decreases (*b* = −0.658, *t*_(14)_ = 1.854, *p* = 0.01). SF-36 VT changes also explained a significant proportion of variance in BDI score changes (*R*^2^ = 0.386, *F*_(1,14)_ = 8.794, *p* = 0.01).

### Functional Connectivity Changes Associated with Tai Chi Intervention

**Figure 1:**
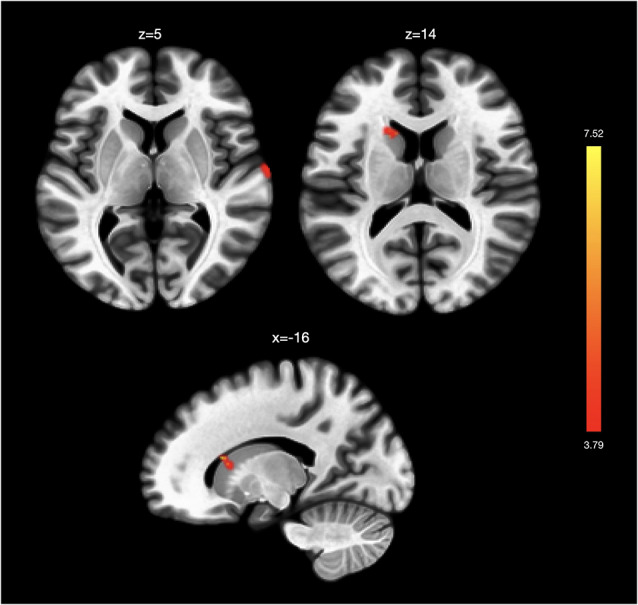
Functional connectivity changes associated with changes in depression. Changes in Beck Depression Inventory (BDI) scores post-intervention were associated with greater functional connectivity between the right anterior insula and right superior temporal gyrus and left caudate. No significant functional connectivity changes were associated with BDI score changes in analyses using the left anterior insula and the left and right posterior insula as seeds.

Regression analyses exploring overall insula-related FC changes following TC found decreased FC between left AIC and left supplementary motor area (SMA; MNI coordinates *x* = −16, *y* = −10, *z* = 64). In our next analyses related to assessing the association between BDI score changes and FC changes using the right AIC as a seed, we found stronger functional connectivity between the right AIC and left caudate and right superior temporal gyrus (Table 2A; Figure 1). Analyses using the left AIC as a seed did not reveal significant left AIC functional connectivity changes associated with BDI score changes pre- and post-intervention. Similarly, we did not find significant functional connectivity changes associated with BDI change scores using the right and left PIC as a seed.

**Table 2:** Seed-to-voxel results shown for seed-to-voxel functional connectivity changes associated with (A) BDI score changes pre- and post-Tai Chi intervention and with (B) VT score changes pre- and post-Tai Chi intervention.

Seed	Brain region	Coordinates (X Y Z)			Cluster size	*z*-score
A. Changes associated with depression improvement
R anterior insula	L Caudate	−16	20	16	92	4.75
R anterior insula	R Superior Temporal Gyrus	68	−12	8	70	3.98
B. Changes associated with vitality improvement
R posterior insula	L Superior Frontal Gyrus	−12	62	56	70	4.07
R posterior insula	L Superior Parietal Gyrus	−26	−46	50	62	4.75

*Anatomical areas were labeled using Automatic Anatomical Labeling*.

We further observed a qualitative shift in the relationship between BDI scores and FC TC. Specifically, although FC prior to the intervention was largely unrelated to BDI scores, numerous significant relationships between insular connectivity and BDI scores emerged afterward. By and large, these relationships were dominated by two patterns: (1) positive correlation between BDI scores and insular FC with sensory and motor areas; and (2) negative correlation between BDI scores and insular FC with attentional and control areas, including middle frontal gyrus and midcingulate cortex (see Supplementary Table S1).

**Figure 2:**
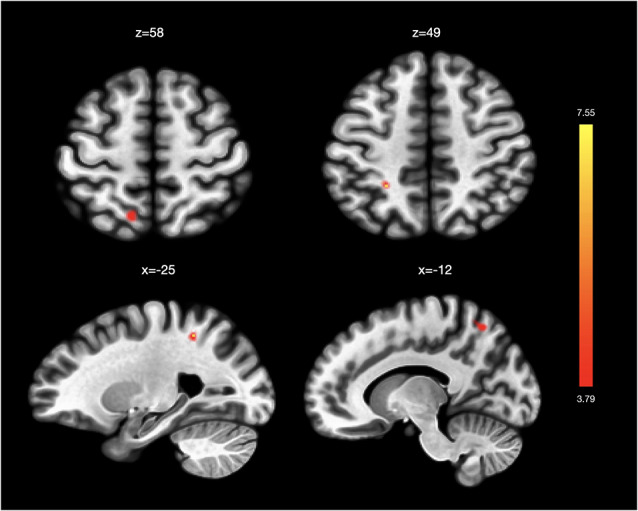
Functional connectivity changes associated with changes in vitality. Changes in SF36-Vitality (VT) scores post-intervention were associated with greater functional connectivity between the right posterior insula and left superior frontal gyrus and left superior parietal gyrus. No significant functional connectivity changes were associated with VT score changes in analyses using the left posterior insula and the left and right anterior insula as seeds.

In analyses related to VT, we found stronger functional connectivity between the right PIC and left superior frontal gyrus and left superior parietal gyrus associated with changes in VT scores post-intervention (Table 2B; Figure 2). We did not find significant functional connectivity changes associated with VT score changes when using the AIC or the left PIC as a seed.

## Discussion

This study investigated the impact of a 10-week TC intervention on subjective reports of depression and vitality in MDD to identify resting-state neural correlates of symptom improvement. We found a significant increase in subjectively reported vitality and a significant decrease in mood symptoms resulting from TC. Moreover, the increase in vitality was associated with the decrease in mood symptoms. However, using our seed-to-voxel rs-FC approach, we were unable to find evidence supporting our hypothesis that improvements in MDD-related symptoms following the intervention would be associated with decreased connectivity between the AIC and mPFC default mode network and enhanced resting connectivity between PIC and AIC. Instead, we demonstrate for the first time that rs-FC changes involving the insula differed depending on whether mood or vitality was examined, further speaking to an overarching model of different roles of AIC and PIC in interoceptive processing in contemplative practices (Farb et al., 2007, 2010, 2013, 2015).

### Symptom Improvement Following Tai Chi

Consistent with prior reports demonstrating TC’s efficacy in treating MDD (Yeung et al., 2012, 2017; Ma et al., 2018), our behavioral findings provide additional evidence for TC as an effective clinical intervention for decreasing depressed mood. Importantly, TC is also effective in increasing the subjective sense of vitality. We further demonstrate that greater vitality increases are associated with mood symptom decreases, suggesting, at least on a behavioral level, a relationship between vitality and mood symptom changes. Osypiuk et al. (2018) recently posited that a link exists between the bodily postures that are trained in TC practice and subsequent elevation of mood and well-being. Our findings may provide some initial support for further investigation of this hypothesis. Although these findings should be interpreted cautiously given the small sample size in our uncontrolled study, our statistical findings are consistent with our hypotheses that TC improves both vitality and depressed mood.

### Functional Connectivity Changes Associated With Symptom Improvement

Overall, in our rs-FC analyses, we found decreased rs-FC between the left AIC and left SMA following the TC intervention. Based on previous work demonstrating, cross-sectionally, increased functional homogeneity in somatomotor areas and decreased functional homogeneity in attentional and control areas (Wei et al., 2014), we speculate that our finding might capture a decoupling between left AIC and left SMA that might reflect a relaxation in the level of control needed to initiate movement in practitioners. In other words, the potential for TC to facilitate movement may be reflected in this decoupling between left AIC and left SMA.

To better examine rs-FC changes related to MDD symptom improvement, we conducted separate analyses. We found stronger FC changes between the right AIC and left caudate and right superior temporal gyrus associated with reduction in depressive symptoms. This result corroborates previous findings of decreased functional connectivity between the right AIC and superior temporal gyrus in depressed patients (Guo et al., 2015) as well as documented blunted responses in the right AIC, superior temporal gyrus, and caudate in depressed patients that may be associated with deficits in cost–benefit based decision making related to effort (Yang et al., 2016). The increased functional connectivity involving the AIC here may suggest TC facilitates mood symptom improvement by strengthening the connection between AIC, which is typically involved in emotional awareness (Craig, 2009), and these regions more involved in effort-based decision making. However, further studies are needed to better unpack whether these neural changes related to symptom improvement in depression associated with TC may themselves relate to effort decisions. Moreover, although our study found involvement of the right superior temporal gyrus and left caudate, these previous studies found involvement of the left superior temporal gyrus and right caudate (Guo et al., 2015; Yang et al., 2016), demonstrating the need to further investigate laterality of functions in these regions. Nevertheless, our right AIC findings point to the potential for AIC-related FC changes in tracking mood symptom improvement following TC as a method for understanding the neural mechanisms by which TC may show clinical efficacy.

Contrary to our findings of right AIC FC changes associated with mood-symptom improvement, we did not find support for left AIC FC changes associated with mood-symptom improvement or for PIC FC changes tracking mood-symptom improvement. Instead, we found that strengthened FC between PIC and sensorimotor areas seemed to be related to improvements in vitality, supporting this brain network’s potential involvement in fatigue and previously proposed roles of the posterior insula in regulating physiological reactivity and homeostatic states (Menon and Uddin, 2010; Deen et al., 2011). Our finding suggest that the vitality effects from TC may be related to changed communication between brain regions involved with the bodily processing (Stephani et al., 2011). This “interoceptive shift” (Payne and Crane-Godreau, 2013) is particularly relevant to consider in light of the active manipulation of *qi* or energy in TC practice, which requires both heightened sensory awareness and a refined intention to “move” energy. Though we cannot conclude from our data what type of bodily processing system may be at work in facilitating this “interoceptive shift,” previous literature has implicated the role of the posterior insula in processing of fatigue (van Duinen et al., 2007). Our findings highlight the need to further explore the potential relationship between “interoceptive shifts,” PIC rs-FC, and improvements in the felt sense of vitality following TC.

Taken together, our seed-based analyses of insula connectivity being associated with depression improvements in MDD is important to consider in light of insular findings from other meditative practices, particularly mindfulness meditation (*not movement*). Previous mindfulness meditation studies have demonstrated that mindfulness practice can impact activity and connectivity of the AIC during interoceptive attention tasks (Farb et al., 2013) and that the AIC is also differentially activated in processing sadness in those trained in mindfulness compared to controls (Farb et al., 2010). These studies utilized task-based fMRI designs comparing recent mindfulness-based stress reduction (MBSR) completers to waitlisted controls in healthy adult populations (Farb et al., 2010, 2013). This study extends these findings by demonstrating that TC, a form of meditative movement, also impacts insula connectivity in a salutary way in resting-state (rather than task-based) functional MRI. It further indicates that adults with MDD also exhibit changes in anterior as well as posterior insular connectivity as a result of meditative movement (e.g., TC) training that is differently associated with mood vs. more somatic symptoms. Farb et al. (2013) conjecture that changes in AIC connectivity following mindfulness practice may reflect a shift toward processing experience according to sensory rather than cognitive aspects, reflective of the fact that mindfulness training involves training of attention toward sensations as they arise without changing them. Because TC training has many similar components to mindfulness training, particularly around the cultivation of mindful attention to bodily sensations and the sensory aspects of emotional experiences, our resting state findings extend Farb’s findings during an interoceptive attention task by suggesting altered moment-to-moment processing of sensory experience even when there isn’t an explicit focus of attention on somatic sensations as is the case during a resting scan.

Nevertheless, although TC practice does involve many commonalities with traditional seated meditation practices (Tsang et al., 2008; Mehling et al., 2011; Klein et al., 2017), particularly in regards to the cultivation of interoceptive awareness (Payne and Crane-Godreau, 2013), one crucial difference between seated meditation and TC is the focus on the active manipulation of energy, or* qi*, in TC practice. Phenomenologically, moving the qi or energy involves not only awareness of subtle bodily sensations but also the intention to actually “move,” or *change*, these sensations to achieve a more positive physiological and psychological state. This process, referred to as a “subjectively experienced interoceptive shift,” (Payne and Crane-Godreau, 2013) may be an experiential correlate of the movement of energy through the body and mind, possibly reflecting an altered relationship to the sensory, proprioceptive, and interoceptive inputs from the body. Our results demonstrate the potential for examining insula-related FC to better unpack these different aspects of TC, especially in relation to their possible clinical efficacy in MDD.

### Limitations and Future Directions

Although we demonstrate insula-related FC changes potentially tracking symptom improvement following TC in distinct manners, it is not clear whether the observed relationships between changes in functional connectivity and MDD symptoms are unique to TC practice. Further studies are needed to develop more nuanced understanding of the impact of TC training on insular connectivity and whether these findings can extend as a framework for examining a neural basis for vitality improvement and mood symptom decrease in other behavioral interventions as well. Prior studies examining the impact of mindfulness training in other populations indicates improvements in vitality post training though neural correlates of the vitality changes were not assessed in these populations (Tavee et al., 2011; Johns et al., 2016). Although this study highlights, at least in a preliminary way, the role of the insula as an interoceptive cortex involved in mediating the subjective sense of vitality as well as depression, further studies are needed to better elucidate this possible interoceptive mechanism and to investigate other mechanisms that may be at play when considering the clinical efficacy of TC for MDD treatment.

To our knowledge, this study is the first to explore the role of insula resting-state functional connectivity in differentially characterizing vitality and mood symptom changes in patients with MDD. Although our study is limited by a small sample size and lack of a control group, our findings suggest depression and vitality are independent constructs mediated by different neural mechanisms, and the relationship between them is complex and not necessarily linear.

Though we have provided several theories that may help in elucidating the implications of our results, additional research is needed to more fully establish how the subjective sense of vitality may be related to neural pathways of “interoceptive shifts.” Future studies directly comparing TC to mindfulness training or other meditative movement practices could be important as a way to further elucidate the effect of the “interoceptive shift” energy manipulation component of TC training, compared to interoception itself, using tasks specific to each. Although we did not explicitly use any behavioral measures of mindfulness or interoceptive awareness, future studies could incorporate those questionnaires to further elucidate the link between mindfulness and meditative movement–specific effects.

Moreover, our study focused on potential differences in functional connectivity changes related to the anterior and posterior insula that tracked depression and fatigue symptom improvement (*via* increased vitality scores) based on prior literature functionally dividing the insula into anterior portions more associated with salience and posterior portions more associated with sensorimotor integration (Cauda et al., 2011). However, other methods of dividing the insula have also been proposed, such as a three-division model of the insula including the mid-insula (Deen et al., 2011), a six-division model from the Brainnetome (Fan et al., 2016), or the entire insula. Future work should investigate different partitions of the insula and how subdivisions may differentially respond to Tai Chi. Importantly, the vitality score used from the SF-36 subscale defines vitality as the opposite of fatigue, so enhanced vitality is indicated by lessened fatigue. Future studies should more thoroughly probe how related vitality and fatigue may be. Are they dissociable constructs or inherently related?

Last, because the population participating in this study were Chinese immigrants, these results may be specific to Chinese patients with MDD. TC was chosen as the intervention for this population because it was hypothesized to be culturally appropriate and perhaps more feasible for a population typically skeptical of mental health interventions (Yeung et al., 2012, 2017). However, TC is gaining popularity in the United States and has potential to be more widely adopted by the general population. Other studies with diverse populations have indeed shown it to be acceptable and effective for those unfamiliar with Chinese culture (Oh et al., 2012; Larkey et al., 2015; Lauche et al., 2016). Future studies should further explore whether these results transcend cultural specificity and can apply to populations or cultures less familiar with TC.

## Conclusion

In conclusion, our study provides preliminary evidence that TC training can therapeutically affect both somatic (vitality) and mood (depression) symptoms in MDD. Mood-related symptoms of MDD have been well studied; however, improvement in fatigue (low vitality) has been an important and largely neglected clinical target in MDD treatment. We demonstrate that insular connectivity may be an important neural mechanism differentially characterizing these symptom changes using resting-state functional connectivity pre and post 10 weeks of TC training. The anatomical specificity in the changes of anterior and posterior insular rs-FC related to depressed mood reductions and vitality increase respectively provides evidence that TC may be an effective behavioral intervention for MDD that can target specific alterations in insula-related sensory processing and integration of sensory cues with affective and external context. These data provide preliminary evidence that mood and fatigue symptoms of depression may be related to different subsystems of the insular cortex. Furthermore, the data suggest that these symptoms are neurobiologically distinct, that fatigue is not merely a by-product of low mood, but rather is an independent symptom. Given that fatigue is not readily alleviated by antidepressants, these findings provide strong evidence for the utility of adding TC as a complementary treatment for MD. Additional study is needed to examine more specifically whether TC uniquely enhances vitality through “interoceptive shifts” resulting from the energy manipulation component of TC training.

## Data Availability Statement

The datasets for this article are not publicly available because they contain information that could be used to identify participants. Requests to access the datasets should be directed to Chloe Zimmerman, chloe_zimmerman@brown.edu.

## Ethics Statement

This study was carried out in accordance with the recommendations of the Institutional Review Board of Massachusetts General Hospital with written informed consent from all subjects. All subjects gave written informed consent in accordance with the Declaration of Helsinki. The protocol was approved by the Massachusetts General Hospital Institutional Review Board.

## Author Contributions

AX and CZ performed the analysis and contributed to the writing of the manuscript. YM acquired the behavioral data and provided critical feedback and revisions on drafts. AY was responsible for initial study design and securing of funding sources for data collection, organized the Tai chi classes, and assisted in feedback on manuscript drafts. SL and CK oversaw the hypothesis generation, analysis, and writing of the manuscript and draft revisions.

## Conflict of Interest

The authors declare that the research was conducted in the absence of any commercial or financial relationships that could be construed as a potential conflict of interest.

## Footnotes

^1^http://neurosynth.org/analyses
