# Supplementary material for: Distinct Insular Functional Connectivity Changes Related to Mood and Fatigue Improvements in Major Depressive Disorder Following Tai Chi Training: A Pilot Study
Source: Front Integr Neurosci. 2020 May 28;14:25. doi: 10.3389/fnint.2020.00025 (PMC7295154; doi:10.3389/fnint.2020.00025)
Supplement: Supplementary file 1 [file Table_1.DOCX]

**Supplementary Table 1.** Seed-to-voxel results shown for seed-to-voxel functional connectivity associated with (a) BDI scores changes pre-Tai Chi intervention and (b) post-Tai Chi intervention. Anatomical areas were labeled using Automatic Anatomical Labeling.

| **Seed** | **Brain region** | **Coordinates (X Y Z)** | | | **Cluster size** | **Peak *z*-score** |
| --- | --- | --- | --- | --- | --- | --- |
|  |  |  |  |  |  |  |
| *A. Changes associated with depression pre- intervention* | | | | | | |
| R posterior insula | R Inferior Temporal Lobe | -8 | -64 | 24 | 377 | z > -8 |
| *B. Changes associated with depression post- intervention* | | | | | | |
| R anterior insula | L Cuneus | -8 | -64 | 24 | 377 | z > -8 |
| R anterior insula | L Middle Frontal Gyrus | -32 | 48 | 18 | 354 | z < -8 |
| L anterior insula | L Angular Gyrus | -38 | -58 | 28 | 115 | 3.80 |
| L anterior insula | R Cerebellum | 36 | -74 | -58 | 115 | 4.08 |
| L anterior insula | L Postcentral Gyrus | -40 | -28 | 56 | 84 | 3.73 |
| L anterior insula | R Hippocampus | 36 | -2 | -16 | 480 | -4.34 |
| L anterior insula | L Midcingulum | -8 | 14 | 34 | 218 | -3.88 |
| L anterior insula | R Superior Temporal Gyrus | 56 | -36 | 24 | 184 | -4.07 |
| L anterior insula | L Insula | -36 | 6 | -6 | 171 | -4.75 |
| L anterior insula | L Cerebellum | -42 | -62 | -28 | 171 | -4.47 |
| L anterior insula | R Middle Frontal Gyrus | 34 | 54 | 28 | 99 | -3.92 |
| L anterior insula | R Middle Frontal Gyrus | 48 | 54 | 10 | 96 | -4.38 |
| L anterior insula | R Midcingulum | 12 | -22 | 38 | 72 | -3.75 |
| L anterior insula | R Middle Frontal Gyrus | 46 | 6 | 56 | 50 | -3.82 |
| R posterior insula | L Middle Frontal Gyrus | -24 | 32 | 40 | 71 | 3.69 |
| L posterior insula | L Precentral Gyrus | -56 | 6 | 44 | 74 | 4.75 |
